# Essentials of Diagnosis

**Published:** 1901-03

**Authors:** 


### Reviews of Books.

---

**Essentials of Diagnosis.** By SOLOMON SOLIS-COHEN, M.D., and AUGUSTUS A. ESHNER, M.D. Second Edition. Pp. 417. London: Henry Kimpton. 1900.

What the object is of arranging a book in the form of question and answer, it is very difficult to see. That there is some demand for it is probable, as there are so many of them. No doubt the method handicaps anyone in writing a work on medicine. But even apart from this there is but little to say in favour of the work. The accounts of nearly everything are meagre, and one description has to do duty for all varieties.

Take thoracic aneurysm, there is no mention of distinction of parts of the aorta affected, and all the symptoms and signs possible from any position are put into one description.

Some of the questions are curious, as "How is emphysema to be distinguished from a pleural effusion?" "How is tuberculous pharyngitis to be distinguished from typhoid fever?"

The essential principle of diagnosis depends on taking the prominent signs and symptoms and arguing from them by exclusion or otherwise to the probable seat of disease. One would, therefore, consider that in a book devoted to the subject that a prominent feature would be the causes of various symptoms; however, in this work that is not the line which is followed. For instance, headache and coma are not treated of at all under those heads, and to find out their importance reference has to be made to numerous diseases in various parts of the book.

If one has made a diagnosis, then by reference to this book it can be seen that the symptoms already discovered have been by others known to exist in that disease; and this is not, as far as diagnosis goes, of much use after the fact. It is hard to see what class of reader will benefit by adding this book to his library.

